# COVID-19 Outcomes and Diabetes Mellitus: A Comprehensive Multicenter Prospective Cohort Study

**DOI:** 10.3390/microorganisms11061416

**Published:** 2023-05-27

**Authors:** Karolina Akinosoglou, Georgios Schinas, Evanthia Bletsa, Magdaline Bristianou, Leonidas Lanaras, Charalambos Michailides, Theodoros Katsikas, Fotios Barkas, Evangelos Liberopoulos, Vasileios Kotsis, Konstantinos Tentolouris, Pinelopi Grigoropoulou, Archontoula Frangou, Dimitrios Basoulis, Zoi Alexiou, Mary Daganou, Clementine Bostantzoglou, Vasiliki Dimakopoulou, Antonia Koutsoukou, Angelos Pefanis, Ioannis G. Baraboutis, Eleni Agelonidou, Nikolaos Tentolouris

**Affiliations:** 1 Department of Internal Medicine, Faculty of Medicine, University of Patras, University Hospital of Patras, 265 04 Patras, Greece; akin@upatras.gr (K.A.); georg.schinas@gmail.com (G.S.); vasilina.dim@gmail.com (V.D.); 2 Department of Internal Medicine, General Hospital of Lamia, 351 00 Lamia, Greece; evabletsa@gmail.com (E.B.); panouharis@gmail.com (M.B.); leolanaras@gmail.com (L.L.); 3 1st Department of Internal Medicine, General Hospital of Athens “G. Gennimatas”, 115 27 Athens, Greece; chmichailidis@gmail.com (C.M.); katsikastheodoros@gmail.com (T.K.); 4 2nd Department of Internal Medicine, Faculty of Medicine, School of Health Sciences, University of Ioannina, 451 10 Ioannina, Greece; fotiosbarkas@gmail.com (F.B.); vaglimp@yahoo.com (E.L.); 5 3rd Department of Internal Medicine, Medical School, Aristotle University of Thessaloniki, General Hospital of Thessaloniki “Papageorgiou”, 564 29 Thessaloniki, Greece; vkotsis@auth.gr; 6 Department of Internal Medicine, General Hospital of Athens “Elpis”, 115 22 Athens, Greece; kostento1@yahoo.com (K.T.); peggigrigo@yahoo.gr (P.G.); atetoka@yahoo.gr (A.F.); 7 1st Department of Internal Medicine, Medical School, National and Kapodistrian University of Athens, Laiko General Hospital, 11527 Athens, Greece; dimitris.bassoulis@gmail.com; 8 General Hospital of Eleusis “Thriasio”, 196 00 Athens, Greece; z_alexiou@yahoo.gr; 9 Intensive Care Unit, General Hospital for Thoracic Diseases “Sotiria”, 115 27 Athens, Greece; mdaganou@hotmail.com (M.D.); medp2011815@med.uoc.gr (C.B.); 10 1st University Pulmonology Clinic and ICU, Medical School, National and Kapodistrian University of Athens, General Hospital for Thoracic Diseases “Sotiria”, 115 27 Athens, Greece; akoutsou@med.uoa.gr; 11 Department of Medicine and 1st Department of Infectious Diseases, General Hospital for Thoracic Diseases “Sotiria”, 115 27 Athens, Greece; pefan1@otenet.gr; 12 Department of Internal Medicine, “Pammakaristos” Hospital, 111 44 Athens, Greece; ioannisbaraboutis@yahoo.gr (I.G.B.); eleni_agelonidou@yahoo.com (E.A.)

**Keywords:** diabetes mellitus, type 2, COVID-19, SARS-CoV-2, disease severity, mortality, in-hospital complications, hypoglycemic agents, DPP4 inhibitor, dipeptidyl-peptidase IV inhibitors

## Abstract

The link between type 2 diabetes (T2D) and the severe outcomes of COVID-19 has raised concerns about the optimal management of patients with T2D. This study aimed to investigate the clinical characteristics and outcomes of T2D patients hospitalized with COVID-19 and explore the potential associations between chronic T2D treatments and adverse outcomes. This was a multicenter prospective cohort study of T2D patients hospitalized with COVID-19 in Greece during the third wave of the pandemic (February–June 2021). Among the 354 T2D patients included in this study, 63 (18.6%) died during hospitalization, and 16.4% required ICU admission. The use of DPP4 inhibitors for the chronic management of T2D was associated with an increased risk of in-hospital death (adjusted odds ratio (adj. OR) 2.639, 95% confidence interval (CI) 1.148–6.068, *p* = 0.022), ICU admission (adj. OR = 2.524, 95% CI: 1.217–5.232, *p* = 0.013), and progression to ARDS (adj. OR = 2.507, 95% CI: 1.278–4.916, *p* = 0.007). Furthermore, the use of DPP4 inhibitors was significantly associated with an increased risk of thromboembolic events (adjusted OR of 2.249, 95% CI: 1.073–4.713, *p* = 0.032) during hospitalization. These findings highlight the importance of considering the potential impact of chronic T2D treatment regiments on COVID-19 and the need for further studies to elucidate the underlying mechanisms.

## 1. Introduction

The outbreak of the COVID-19 pandemic has put immense pressure on the global healthcare system and emphasized the importance of understanding the interplay between comorbidities and severe acute respiratory syndrome coronavirus 2 (SARS-CoV-2). Except for the most prevalent and well-studied cardiovascular diseases (CVDs), with hypertension (HTN) found in approximately 30% of infected patients, diabetes mellitus (DM), mainly type 2 (T2D), affects approximately 9.7% of all individuals with COVID-19 [1,2]. Even though such a rate is comparable to that in the general population, when it is compared between severe and non-severe COVID-19 disease, T2D was even three-fold higher in patients who required admission to intensive care units (ICUs) than those following a mild course of SARS-CoV-2 infection [1,3]. In addition, the mortality rate among COVID-19 patients with T2D is nearly double that of those without diabetes [4]. Globally, the prevalence of diabetes among all patients with COVID-19 varies among reports, ranging from 7 to 21% [2]. Data deriving from a recent review and meta-analysis showed that the pooled prevalence of diabetes in stratified COVID-19 groups was 14.7% among confirmed cases, 10.4% among non-hospitalized cases, 21.4% among hospitalized cases, 11.9% among non-severe cases, 28.9% among severe cases, and 34.6% among deceased individuals, respectively [5]. Hence, T2D was identified as a major risk factor for severe illness and poor outcomes from COVID-19 [6,7,8,9,10,11].

Despite ongoing research, the precise connection between T2D and COVID-19 severity or susceptibility is still not fully understood. Currently, there are few conclusive data to suggest that diabetes increases the risk of contracting COVID-19 [12]. Hyperglycemia can potentiate SARS-CoV-2 infectivity and support accelerated viral proliferation. Hyperglycemia seems to directly increase SARS-CoV-2 replication in vitro, and glycolysis can in turn sustain viral replication [13]. On the other hand, impaired immune response to SARS-CoV-2, due to chronic hyperglycemia and the low-grade chronic inflammation associated with T2D, represents another parameter contributing to worse outcomes [14]. DM can weaken the immune system through various mechanisms, including poor chronic glycemic control as well as acute hyperglycemia [15,16]. Hyperglycemia upon admission has been proven to be a major predictor of death and severe sequelae in COVID-19 [17]—although the same does not apply to stress hyperglycemia [18]; however, the SARS-CoV-2 infection can induce hyperglycemia and reveal newly onset diabetes [19]. In addition, oxidative stress and the production of adhesion molecules that mediate inflammation in the tissue, in combination with the binding of SARS-CoV-2 to ACE-2 receptors expressed in the cells of the pancreas, adipose tissue, and intestine might cause alterations in glucose metabolism and contribute to the augmented severity of COVID-19 [20,21,22]. On the other hand, patients with T2D constitute a highly heterogeneous population in terms of the aforementioned comorbidities, such as CVD and HTN, but also obesity, or diabetic complications. Obviously, each of them alone could increase the risk of a severe course of COVID-19 and drive outcomes [23,24], as also shown in previous studies as per the effect of co-morbidities on sepsis in diabetic patients [25].

Thus, maintaining good blood sugar control is crucial in mitigating the severity of COVID-19 [26]. To effectively manage DM and cater to the individual needs of patients, multiple therapeutic strategies have been developed. Common treatments for day-to-day management include antidiabetic medications, such as glucagon-like peptide-1 (GLP-1) receptor agonists and dipeptidyl peptidase 4 (DPP4) inhibitors, due to their efficacy, ease of administration, and favorable safety profiles. Insulin remains the primary treatment for long-standing DM, particularly for insulin-dependent patients, and requires special attention. Most diabetes drugs can be continued during COVID-19, but insulin is often preferable in severe conditions [27]. Investigating the various T2D treatment regimens, including insulin, metformin, sodium-glucose transport protein 2 (SGLT2) inhibitors, DPP4 inhibitors, sulfonylureas, and GLP-1 receptor agonists, can be performed by examining their influence on immune pathways that mediate the viral replication or host response. However, the mechanisms of action of these treatment options are vastly different, and the impact of each pathway on ameliorating or exaggerating COVID-19 severity may not have been fully explored or investigated. Additionally, the complex interplay between immune-mediated pathways and disease severity can be difficult to establish. For example, some T2D treatments, such as insulin, directly regulate the immune system [28], while others, such as SGLT2 inhibitors and metformin, may impact the gut microbiome and indirectly modulate the immune response [29].

The strong association between T2D and COVID-19 severity underscores the urgent need for a more comprehensive understanding of this relationship. The precise underlying mechanisms remain unknown, and it is unclear whether the increased incidence of complications in individuals with DM results from the interplay between the virus, the immune system, diabetes itself and antidiabetic medication, or related co-morbidities. We aimed to report the experience of COVID-19 outcomes in patients with T2D in the context of a national registry and investigate the potential associations with antidiabetic medications.

## 2. Materials and Methods

### 2.1. Study Design and Data Collection

This was a multicenter prospective cohort study designed to assess the outcomes of COVID-19 in patients with T2D and explore associations between T2D treatment regiments and adverse clinical outcomes in Greece during the third wave of the pandemic (February–June 2021). Adult SARS-CoV-2 positive patients with T2D using polymerase chain reaction (PCR) and requiring hospitalization were included in this study in the context of a national registry. Epidemiologic and clinical characteristics were recorded, including age, sex, body mass index (BMI), and presence of comorbidities, i.e., hypertension (HTN), cardiovascular disease (CVD), chronic obstructive pulmonary disease (COPD), chronic kidney disease (CKD)/end-stage renal disease (ESRD), cancer, and co-medications, e.g., (statins, ACE-inhibitors/ARB). This study also collected data on the types of medication used for the chronic management of T2D, including insulin, metformin, sulfonylureas, DPP4 inhibitors, GLP-1 receptor agonists, and SGLT2 inhibitors. Laboratory values and outcomes of interest, i.e., in-hospital death, disease progression to acute respiratory distress syndrome (ARDS), and ICU admission or adverse events including acute kidney injury (AKI), acute cardiac injury (ACI), acute liver injury (ALI), shock, and bacterial lung superinfection were also recorded. This study was approved by the Institutional Review Board 2716/02.02.21 and was conducted in accordance with the Declaration of Helsinki, Principles of Good Clinical Research Practice and Regulations on General Data Protection.

### 2.2. Statistical Analysis

The statistical analysis was conducted using SPSS 28 (IBM, USA) and R (version 3.6.3) software packages. Descriptive statistics are presented as medians and first and third quartiles’ range (median (IQR)) for quantitative variables, and absolute and relative frequencies for qualitative variables. For the inferential part of the analysis, we used the Shapiro–Wilk test to assess the normality of continuous variables, and when the data were not normally distributed, we used non-parametric tests such as the Mann–Whitney U-test to compare two independent groups for quantitative variables. The T-test was used for normally distributed variables. The frequencies of qualitative variables were compared using the chi-square (χ2) test and Fisher’s exact test.

We developed multivariate logistic regression models to predict in-hospital survival, progression to ARDS, and ICU admission, adjusting for potential confounding variables. Predictor variables were chosen based on their known or suspected associations with adverse clinical outcomes. Male sex, age, BMI, and DM-relevant information were added to the models. Chronic T2D treatment regimens were examined separately. Results are expressed as odds ratios (OR) with 95% confidence intervals (CI) for univariate analysis and adjusted ORs with 95% CIs for multivariate analysis.

We used Cox proportional hazards regression to assess the association between prior DPP4 inhibitor use and death within 28 days of hospitalization, adjusting for potential confounders. Univariable analysis identified the factors predicting in-hospital death, and the proportional hazards assumption was visually confirmed. The multivariable analysis included candidate independent variables with a regression parameter which was significantly different from zero at *p* < 0.05, or those deemed necessary, including age, sex, and BMI. Model goodness of fit and assumptions were assessed. Results were reported as hazard ratios (HRs) with 95% confidence intervals (CI) and *p*-values.

For the analysis of in-hospital complications, we initially performed chi-square tests to assess potential associations. We then calculated the symmetric measures, including Pearson’s R to evaluate the strength of these associations. Following this, we conducted the logistic regression analysis to explore the influence of possible risk factors on the respective complications, while adjusting for age and sex.

All statistical analyses were conducted using appropriate methods, and a two-sided *p*-value of less than 0.05 was considered statistically significant.

## 3. Results

### 3.1. Population Characteristics

A total of 354 T2D patients with COVID-19 were included in this study (Table 1). Male sex represented 53.1% of the sample, and the median age was 70 years (IQR: 62–79). The majority (92.1%) were of Greek nationality with a median BMI of 28.3 kg/m^2^ (IQR: 26.22–31.55). The prevalence of tobacco use was distributed as follows: never (44.1%), former (33.9%), and current users (13.6%). The median HbA1C level was 7% (IQR: 6.4–7.8). Prevalent comorbidities included HTN (72.6%), chronic heart failure (CHF) (14.6%), and COPD (12.4%). Among the COVID-19 complications, ARDS was present in 24.3% of patients, and bacterial superinfection was present in 23.9%. The in-hospital mortality rate was 18.6%. Corticosteroids and antibiotics were the most commonly administered COVID-19 treatments, at 85.6% and 84.4%, respectively. Metformin (42.4%) and DPP4 inhibitors (37.9%) were the predominant T2D chronic treatment regimens.

### 3.2. Mortality

In this study, 276 patients with COVID-19 survived their hospitalization, while 63 patients died. Survivors had a significantly lower median age (69 years) compared to those who died (80 years, *p* < 0.001). The prevalence of tobacco use was higher among survivors (15.2%) compared to non-survivors (6.3%), whereas former smokers were more prevalent in the in-hospital death subgroup, constituting almost half of the group (47.6%). Patients who died were also more likely to have micro-/macrovascular complications of T2D, including ischemic heart disease (IHD) (38.1% vs. 20.3%, *p* = 0.003), cerebrovascular accidents (CVAs) (21.7% vs. 5.2%, *p* < 0.001), and CKD/ESRD (32.8% vs. 12.7%, *p* < 0.001). Additionally, they were more likely to have CHF (24.2% vs. 12.9%, *p* = 0.024), malignancy (14.8% vs. 5.9%, *p* = 0.028), and chronic cognitive deficit (13.1% vs. 5.5%, *p* = 0.046) compared to survivors, and experience COVID-19 complications such as ARDS (77.8% vs. 12.7%, *p* < 0.001), AKI (33.3% vs. 6.5%, *p* < 0.001), ACI (34.9% vs. 3.3%, *p* < 0.001), shock (40.3% vs. 1.1%, *p* < 0.001), bacterial superinfection (57.1% vs. 17%, *p* < 0.001), thrombotic event (25.4% vs. 6.2%, *p* < 0.001), and hemodialysis (4.8% vs. 0.7%, *p* = 0.047). The previous use of DPP4 inhibitors as part of their chronic DM treatment regimen was associated with an increased risk of in-hospital death (50.8% vs. 35.9%, *p* = 0.028) compared to non-use (Table 2).

**Figure 1:**
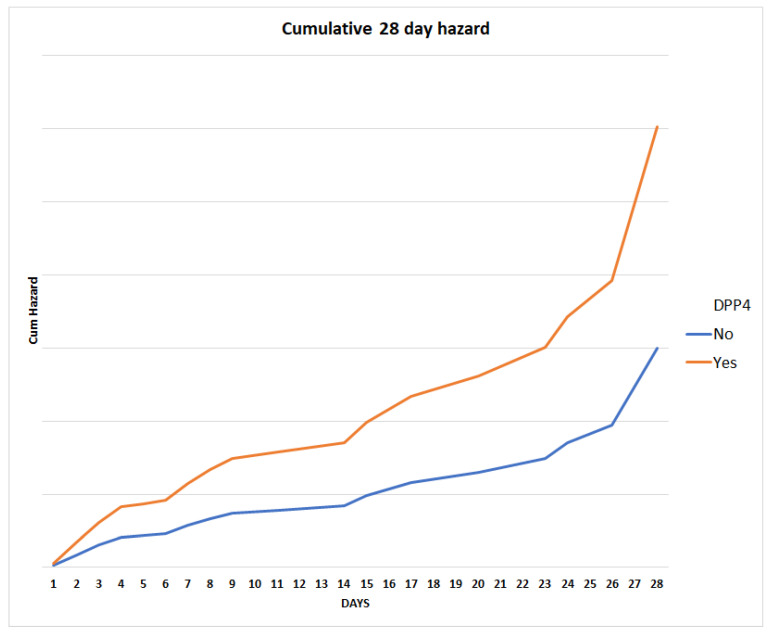
Twenty-eight-day mortality cumulative hazard for the constructed model, stratified for prior DPP4 inhibitor use.

We conducted a Cox regression analysis to further investigate the factors associated with 28-day mortality in our patient population. Our results suggest that male sex (adjusted hazard ratio (HR) = 2.468, 95% CI: 1.222–4.984, *p* = 0.012), age (adj. HR = 1.058, 95% CI: 1.019–1.099, *p* = 0.003), chronic neurological disease (adj. HR = 4.262, 95% CI: 1.759–10.322, *p* = 0.001), and prior DPP4 inhibitor use (HR = 2.014, 95% CI: 1.082–3.750, *p* = 0.027) were significantly associated with an increased risk of mortality in the first 28 days (Figure 1, Supplementary Table S2). Moreover, years since T2D diagnosis were found to be inversely associated with the 28-day mortality risk (adj. HR = 0.948, 95% CI: 0.905–0.992, *p* = 0.021).

### 3.3. ICU Admission

A minority (16.4%) of patients required ICU admission during the course of their hospitalization. Compared to patients without a need for the ICU level of care, those admitted to the ICU were more likely to be male (69% vs. 50%, *p* = 0.008) and less likely to have HTN (57.9% vs. 75.4%, *p* = 0.007). Their median hospital length of stay (LOS) was more than double (23 days, IQR 15–32) that of those not requiring ICU admission (10 days, IQR 6–15) (*p* < 0.001) and their in-hospital mortality rate was significantly higher (46.6% vs. 10.1%, *p* < 0.001). In terms of COVID-19 complications, patients admitted to the ICU had significantly higher rates of ARDS, ALI, bacterial superinfection, shock, and thrombotic events (all *p* < 0.05). Additionally, prior treatment with DPP4 inhibitors proved to be significantly higher in ICU-admitted patients compared to non-ICU admitted patients (50% vs. 35.5%, *p* = 0.037) (Table 2).

The multivariate analysis revealed that the chronic use of DPP4 inhibitors was significantly associated with increased odds of ICU admission (adj. OR = 2.524, 95% CI: 1.217–5.232, *p* = 0.013). Male sex (adj. OR = 2.278, 95% CI: 1.022–5.075, *p* = 0.044) and years living with diabetes (adj. OR = 0.918, 95% CI: 0.858–0.982, *p* = 0.013) were also found to be significantly associated with ICU admission. Other patient characteristics, including age, BMI, HbA1c, and comorbidities such as IHD, and CKD, were not significantly associated with ICU admission neither in univariate nor in multivariate analysis (Supplementary Table S1).

The use of ARBs/ACE inhibitors was associated with a lower risk of ICU admission (OR = 0.537, 95% CI 0.302–0.954, *p* = 0.034), although the association became non-significant after adjusting for other covariates (adj. OR = 0.699, 95% CI 0.259–1.888, *p* = 0.480). The same applies to HTN, which was associated with a lower risk of ICU admission (OR = 0.448, 95% CI 0.249–0.808, *p* = 0.008) in univariate analysis; however, following adjustment for pertinent confounders, the association became non-significant (adj. OR = 0.489, 95% CI 0.179–1.340, *p* = 0.164).

When the inclusion of DM treatment modalities other than DPP4 inhibitors was tested in the final model constructed, it was revealed that chronic insulin treatment was independently associated with decreased odds of ICU admission (adj. OR 0.259, 95% CI 0.074–0.909, *p* = 0.035).

### 3.4. Progression to ARDS

We compared 268 patients and 86 patients without and with ARDS in this cohort, respectively. There were no significant differences between subgroups with regard to age at T2D diagnosis, duration of diabetes, insulin use, HbA1c levels, and micro-/macrovascular complications. However, patients with ARDS were more likely to have been prescribed DPP4 inhibitors prior to their admission for COVID-19 (*p* = 0.008). In-hospital death was significantly higher in the ARDS group compared to the non-ARDS group (*p* < 0.001). Additionally, AKI (*p* < 0.001), acute cardiac injury (*p* < 0.001), ALI (*p* < 0.001), shock (*p* < 0.001), bacterial superinfection (*p* < 0.001), and thrombotic events (*p* = 0.006) were all significantly more common in the ARDS group (Table 2).

In both the univariate analysis and the multivariate model, the use of DPP4 inhibitors was significantly associated with an increased risk of developing ARDS (OR = 1.945, 95% CI: 1.189–3.183, *p* = 0.008; adj. OR = 2.507, 95% CI: 1.278–4.916, *p* = 0.007). Male sex, older age, T2D duration, and a history of cerebrovascular disease were also found to be significant risk factors for ARDS. Other factors, including other antidiabetic medications, BMI, and comorbidities, did not show a significant association with ARDS (Supplementary Table S1).

### 3.5. Acute Kidney Injury (AKI)

The results of the crosstabulation analysis suggest that CKD, metformin, and DPP4 inhibitor treatment are significantly associated with acute kidney injury in COVID-19 patients with diabetes. The age- and sex-adjusted multivariate analysis results revealed that age and CKD were significant predictors of acute kidney injury. Specifically, the odds of developing acute kidney injury increased by 4.1% for each one-year increase in age, and patients with CKD had 5.98 times higher odds of developing AKI compared to those without CKD. However, male sex and metformin use were not found to be significant predictors. In the model for DPP4 inhibitor use, age and CKD were again found to be significant predictors, while DPP4 inhibitor use was not.

### 3.6. Acute Cardiac Injury (ACI)

Chronic kidney disease (CKD) appears to be the strongest risk factor for ACI (?), with a significant association between these variables (Pearson chi-square 17.0, *p*-value <0.001). Patients with IHD and CHF are also at an increased risk for ACI (*p* < 0.001 for both). The adjusted logistic regression model identified age (*p* = 0.009), prior statin use (*p* = 0.025), and remdesivir use (*p* = 0.026) as significant predictors of ACI. The adjusted odds ratios (ORs) for significant predictors of ACI were as follows: for age, the OR was 1.061 (95% CI: 1.014–1.109); for previous statin use, the OR was 2.887 (95% CI: 1.139–7.317); and for Remdesivir use, the OR was 0.371 (95% CI: 0.155–0.887).

### 3.7. Thromboembolic Events

The results show that the use of DPP4 inhibitors and the history of cerebrovascular accident were significantly associated with an increased risk of thrombotic events or acute arterial embolism (*p* = 0.023 and *p* = 0.031, respectively). The logistic regression analysis revealed that the age, prior DPP4 inhibitor use, and a history of stroke were significantly associated with an increased risk of developing thromboembolic events. The DPP4 inhibitor use was found to be a significant predictor of thromboembolism during hospitalization, with an adjusted OR of 2.249 (95% CI: 1.073–4.713) and a *p*-value of 0.032.

## 4. Discussion

We aimed to study the patterns and outcomes of COVID-19 infection on patients with T2D, and explore the potential associations with co-morbidities and antidiabetic medications. Importantly, we found that DPP4 inhibitors were independently associated with in-hospital adverse events, including progression to ARDS, thromboembolic events, need for ICU, and in-hospital mortality.

COVID-19 patients with preexisting diabetes have been previously reported to have elevated rates of hospital admission, severe pneumonia, and mortality [2,30,31]. Early reports coming from data derived from the CDC in early 2020 showed that the prevalence of diabetes in patients with COVID-19 was 6% for non-hospitalized patients, 24% for hospitalized non-ICU patients, and 32% for hospitalized ICU patients [32]. These data were consistent with those of the Chinese CDC reporting a predominance of diabetic patients among ICU admissions [33]. An approximately 3.5- and a 2-fold increase in hospital COVID-19-related deaths was reported in patients with T1D and T2D, respectively, compared to patients without diabetes in various cohorts [34,35,36]. In our cohort, the need for ICU and in-hospital mortality was reported in 16.5 and 18% of patients with T2D, similarly to previous authors [37] who have identified diabetes as an independent factor for COVID-19 mortality [38]. In line with these findings, a retrospective study from the Massachusetts General Hospital noted higher rates of ICU admission, mechanical ventilation, and death in patients with diabetes within 14 days of admission, [2]. Several meta-analyses came to confirm these findings, across various variant surges demonstrating that a history of diabetes increases the risk of severe infection by between 2- and 2.8-fold, and the risk of COVID-19-associated death by 1.8–3.2-fold [3,12,39,40,41,42,43,44].

Similarly to others, we also found that other risk factors for poor outcomes have included male sex, older age, and cardiovascular or renal disease [45,46]. Non-survivors suffered an increased rate of complications including ARDS, CVA, AKI, ACI, or shock in our cohort. The role of diabetes micro/macro-complications as well as accompanying co-morbidities has been discussed in the past [25]. In a previous large prospective cohort study, also conducted within the same national grounds comparing sepsis outcomes between diabetic and non-diabetic populations, both various adverse events and mortality were comparable, when matched for age, sex, co-morbidities, and disease severity scores [25]. In patients with sepsis and multiple accompanying co-morbidities, it appears that the latter, rather than diabetes per se, drive outcomes after the adjustment of potential confounders [25,47].

Obesity is an independent risk factor for severe COVID-19 illness and the effects of uncontrolled T2D and obesity may be additive [37]. Patients with T2D and obesity are thought to have a higher risk of developing complications from COVID-19 due to underlying chronic inflammation affecting glucose regulation and insulin sensitivity. Glycemic control is pivotal since mortality has been higher among those with a higher HbA1c at baseline [46]. Propensity score-matched cohorts showed that mortality was significantly lower in well-controlled versus poorly controlled T2M, which is also associated with an increased rate of complications [2,48]. However, we found no association with levels of Hb1Ac and poor outcomes in our cohort, in line with data from the prospective Coronavirus and Disease Outcome study reporting no association between glycemic control and mechanical ventilation or death within 7 days of admission [2]. This observation was also confirmed by a retrospective study from New York, even though the need for pre-admission insulin use conferred higher odds of death [49]. It appears that, although the short-term duration of glycemic control, as reflected in variability in glucose levels upon admission and during hospitalization, may play a role in outcomes among the critically ill [50], this is not the case for long-term glycemic regulation, since HbA1c levels do not necessarily seem to affect mortality [51]. As in many studies, most available data do not distinguish between T1D and T2D or disease severity, and conclusions need to be examined with caution.

We also examined the role of antidiabetic therapy in the outcomes of T2D patients with COVID-19. Glucose-lowering agents have been reported to have multiple effects on COVID-19 outcomes [12]. We found that prior DPP4 inhibitor use was associated with increased odds of in-hospital death, need for ICU, the occurrence of thromboembolic events, and acute kidney injury. DPP4, originally known as ‘T-cell antigen CD26’, is expressed in many immune cells and regulates their function [52]. Due to the high affinity between human DPP4 and the spike (S) receptor-binding domain of SARS-CoV-2, it was assumed that the virus might be able to use the DPP4 enzyme as a functional receptor to gain entry into the host, as modelled in silico [53,54]. However, DPP4 inhibitors also exert anti-inflammatory effects, which could be beneficial in patients exposed to cytokine storms due to COVID-19 [55,56]. Previous studies have shown that DPP4 inhibitor sitagliptin could decrease the levels of pro-inflammatory markers such as tumor necrosis factor-α (TNF-α) and interleukin-6 (IL-6) [57,58]. However, when observational (mostly retrospective) studies compared clinical outcomes in DPP4 inhibitor users vs. non-users among diabetes patients with COVID-19, the overall results regarding the risk of progression towards more severe forms of disease and mortality were heterogenous. New expectations rose when recent reports revealed significant reductions in admissions to ICU and mortality in DPP4 inhibitor users of up to 24% [59,60,61,62,63], with several authors suggesting that targeting DPP4 could be a pharmacologically important strategy for treating patients suffering from severe respiratory diseases related to coronaviruses and COVID-19 [64,65]. However, in line with our results, other authors identified an almost 4-fold increased risk of ICU admission and signs of an increased risk of death in this population [66,67]. A recent meta-analysis found that DPP4 inhibitor use was associated with statistically significant higher hospitalization risk (RR:1.44, *p* < 0.001) and higher risk of ICU admissions and/or mechanical ventilations vs. non users (RR:1.24, *p* < 0.02) [68]. Of note, several other studies, mainly retrospective cohorts and subsequent meta-analysis, suggest no harm or benefit of DPP4 inhibitors administration in T2D COVID-19 patients [69,70]. The reason for these discrepancies remain elusive and the design of current studies precludes safe conclusions, while mixed population and individual genomic and transcriptomic signatures could be the reason for these diverse results. Another explanation could lie in the fact that the administration of DPP4 inhibitors comes later in the path of glucose lowering medications, following metformin, SGLT-2, or GLP-1 receptor agonists, and hence, these patients may already represent a population of progressed metabolic disease and complications. Therefore, in-depth studies are still warranted to identify the potential survival benefits associated with the usage of DPP4 inhibition in patients with or without diabetes mellitus and COVID-19.

No other antidiabetic medication was associated with outcomes in this population. Our understanding of metformin and how it mitigates COVID-19 severity remains elusive, as previous studies conferred contradictory results [32,71,72]. GLP-1 receptor agonists seem to have anti-inflammatory effects in animal models and reduce inflammation and biomarkers in patients with T2D nonetheless, there are insufficient data as to their use in critically ill or COVID-19 [73]. Controversial results with insulin and SGLT-2 inhibitors were also reported. A small study showed that insulin infusion was beneficial in achieving glycemic targets, hence ensuring a better COVID-19 outcome [74]; however, larger observational studies showed association with increased mortality [75,76]. Similarly, SGLT-2 inhibitors, may prevent release of pro-inflammatory cytokines and increase the production of angiotensin 17, thus protecting against the development of ARDS [77]; however, at the same time, they have been reported to increase the risk of AKI and hemodynamic instability during acute systemic infection [73].

This study has several limitations that should be considered when interpreting the results. First, this study is observational in nature, which may introduce bias or confounding due to unmeasured or unknown factors. Secondly, since the investigation was carried out across several centers, patient population heterogeneity and different practices might arise and impede the generalizability of the findings to other healthcare environments. Moreover, this study was carried out during the third wave of the pandemic, during which the delta variant prevailed. It remains uncertain whether the omicron surge would exhibit the same behavior in T2D patients. Of note, the beginning of 2021 was marked by the initiation of vaccination, primarily in the elderly, followed by high-risk groups. In Greece, people with DM were included in late May 2021, and even though such information was not available, it seems unlikely that the small proportion of older individuals with T2D and early vaccination drove the outcomes of this study. Moreover, even though the category of drug was recorded, no differentiation was made upon different regimens within the same class. It is possible that, e.g., various DPP4 inhibitors pose different effects, similarly to their variable antidiabetic potency. In spite of these caveats, multiple measures were employed to mitigate potential confounding and ensure data accuracy. Firstly, the cohort design facilitated the identification of potential confounding factors and the adjustment of their effects during the statistical analysis. This study was restricted to T2D patients, in order to ensure sample homogeneity, since T1D and T2D differ in underlying pathophysiology, and often in the duration of metabolic disease and the occurrence of micro- or macro-complications. Moreover, an extensive data extraction and validation process was implemented at each participating center to ensure data accuracy and completeness. Despite these measures, it is possible that residual confounding or other sources of bias may still be present in this study, and the results should be interpreted with caution.

## 5. Conclusions

We aimed to explore outcomes and uncover respective associations in patients with T2D and COVID-19 in the context of a national registry. By assessing the prevalence of comorbidities and outcomes among these patients, healthcare providers can gain invaluable insights, enabling them to identify high-risk individuals and adapt treatment approaches as needed. Shaping public health policies and decisions regarding resource allocation, as a deeper understanding of T2D, its treatments, and COVID-19, may facilitate the creation of more effective prevention strategies and patient management plans that can enrich clinical decision making and contribute to improved healthcare outcomes during an ongoing global health crisis. The DM and COVID-19 relationship is bidirectional and promotes a syndemic approach, allowing for multidisciplinary management that can ensure the best outcomes both during acute disease, as well as post-COVID-19 [78,79,80]. Examining each treatment regimen in terms of eventual disease outcome may provide critical insights and raise important questions regarding the need to maintain or withhold certain antidiabetic medications during acute disease. However, it is important to note that these observations are not founded on physiologic principles and should not be used to make clinical decisions on treatment choice or induce adjustments based on prevention tactics. Future larger prospective interventional studies are needed in order for safe conclusions to be drawn.

## Figures and Tables

**Table 1:** Population characteristics.

	All (*n* = 354)
**Male sex, n (%)**	188 (53.1)
**Age (y) (IQR)**	70 (62–79)
**Greek nationality (%)**	326 (92.1)
**BMI (kg/m^2^) (IQR)**	28.3 (26.22–31.55)
***Tobacco use (categories)***	
**Never (%)**	156 (44.1)
**Former (%)**	120 (33.9)
**Current (%)**	48 (13.6)
***Type 2 DM information***	
**Age at time of DM diagnosis (years) (IQR)**	60 (51–67)
**Diabetes duration (y) (IQR)**	10 (5–16)
**Insulin use in the last 2 years (%)**	23 (6.6)
**HbAc (%) (IQR)**	7 (6.4–7.8)
***Micro-/macrovascular DM complications (n) (%)***	
**Ischemic heart disease (IHD)**	80 (22.9)
**Cerebrovascular accident (CVA)**	28 (8.1)
**CKD/end-stage renal disease (ESRD)**	56 (16)
**Revascularization of any artery**	29 (8.3)
***Comorbidities (n) (%)***	
**Hypertension (HTN)**	254 (72.6)
**Congestive heart failure (CHF)**	51 (14.6)
**Malignancy**	26 (7.4)
**Liver disease**	7 (2)
**Chronic obstructive pulmonary disease (COPD)**	43 (12.4)
**Chronic cognitive deficit**	23 (6.6)
***Regular medication (n) (%)***	
**ACE-inhibitors/ARBS**	192 (55)
**Statins**	198 (56.6)
**Immunosuppressive drugs**	21 (6)
***In-hospital outcomes***	
**ICU admission *n (%)***	58 (16.4)
**In-hospital death *n (%)***	63 (18.6)
**Hospital LOS, median (IQR)**	11 (7–17)
**ICU LOS, median (IQR)**	13 (10–20)
***COVID-19 Complications n (%)***	
**Acute respiratory distress syndrome (ARDS)**	86 (24.3)
**Acute kidney injury (AKI)**	43 (12.1)
**Acute cardiac injury (ACI)**	31 (8.8)
**Acute liver injury**	28 (8)
**Shock**	28 (8)
**Bacterial superinfection**	84 (23.9)
**Thrombotic event**	34 (9.6)
**Hemodialysis**	5 (1.4)
**Diabetic ketoacidosis (DKA)**	4 (1.1)
***COVID-19 Treatment modalities (%)***	
**Remdesivir**	194 (55.1)
**Corticosteroids**	303 (85.6)
**Antibiotics**	298 (84.4)
**Tocilizumab**	63 (18)
**Anakinra**	11 (3.1)
**Oxygen therapy**	321 (90.9)
**High flow nasal cannula (HFNC)**	31 (8.9)
**Non-invasive ventilation (NIV)**	93 (26.5)
***Type 2 DM chronic treatment regimen (%)***	
**Antidiabetic tablets**	301 (86.2)
**Metformin**	147 (42.4)
**Sulfonylureas**	35 (10)
**Dipeptidyl peptidase 4 (DPP4) inhibitors**	134 (37.9)
**Glucagon-like peptide-1 receptor agonists (GLP1-RA)**	38 (11)
**Insulin**	73 (20.8)
**Insulin plus GLP1-RA**	14 (4)
**Sodium-glucose co-transporter-2 inhibitors (SGLT2i)**	52 (14.7)

**Table 2:** In-hospital outcomes.

Population Characteristics	In-Hospital Death			ICU Admission			ARDS		
	No (*n* = 276)	Yes (*n* = 63)	*p* Value	No (*n* = 296)	Yes (*n* = 58)	*p* Value	No (*n* = 268)	Yes (*n* = 86)	*p* Value
Male sex, n (%)	142 (51.4)	35 (55.6)	0.556	148 (50)	40 (69)	0.008	137 (51.1)	51 (59.3)	0.186
Age (y)	69 (59–76)	80 (70–84)	<0.001	71 (62–81)	69 (59–77)	0.193	69 (61–78)	73 (62–82)	0.15
Greek nationality (%)	254 (92)	62 (98.4)	0.093	270 (91.2)	56 (96.6)	0.283	243 (90.7)	83 (96.5)	0.08
BMI (kg/m^2^)	28.3 (26.5–32.3)	27.7 (24–29.74)	<0.001	28.1 (26–31.8)	29.3 (27.3–31.2)	0.18	28.2 (16.2–31.9)	28.55 (26.3–30.8)	0.704
Tobacco use (categories) (%)			0.021			0.046			0.219
Never	132 (47.8)	23 (36.5)		137 (46.3)	19 (32.8)		121 (45.1)	35 (40.7)	
Former	88 (31.9)	30 (47.6)		94 (31.8)	26 (44.8)		84 (31.3)	36 (41.9)	
Current	42 (15.2)	4 (6.3)		37 (12.5)	11 (19)		37 (13.8)	11 (12.8)	
Type 2 DM information									
Age at time of DM diagnosis (y)	58 (50–66)	64 (58–75)	<0.001	60 (51–67)	59 (51–70)	0.786	59 (50–66)	61 (54–70)	0.3
Diabetes duration (y)	9 (5–16)	10 (6–19)	0.276	10 (6–16)	8 (5–11)	0.022	10 (5–16)	9 (6–15)	0.256
Insulin w/in 2 years	16 (5.9)	7 (11.5)	0.158	19 (6.6)	4 (6.9)	1	17 (6.5)	6 (7.1)	0.854
HbA1c (%)	7 (6.4–7.8)	7 (6.5–8.4)	0.345	7 (6.4–7.8)	7 (6.5–8)	0.408	7 (6.4–7.8)	7 (6.5–8)	0.559
Micro/Macrovascular DM complications (%)									
Ischemic heart disease (IHD)	55 (20.3)	24 (38.1)	0.003	66 (22.7)	14 (24.1)	0.809	55 (20.9)	25 (29.1)	0.118
Cerebrovascular accident (CVA)	14 (5.2)	13 (21.7)	<0.001	26 (9)	2 (3.6)	0.280	19 (7.1)	9 (10.5)	0.277
CKD/end-stage renal disease (ESRD)	35 (12.7)	20 (32.8)	<0.001	48 (16.3)	8 (14)	0.665	39 (14.6)	17 (20.2)	0.219
Revascularization of any artery	21 (7.7)	6 (9.7)	0.606	24 (8.2)	5 (8.8)	0.797	21 (7.9)	8 (9.3)	0.658
Comorbidities (%)									
Hypertension (HTN)	199 (72.9)	46 (74.2)	0.835	221 (75.4)	33 (57.9)	0.007	198 (74.7)	56 (65.9)	0.112
Congestive heart failure (CHF)	35 (12.9)	15 (24.2)	0.024	46 (15.8)	5 (8.8)	0.172	38 (14.2)	13 (15.3)	0.838
Malignancy	16 (5.9)	9 (14.8)	0.028	21 (7.2)	5 (8.8)	0.678	16 (6)	10 (11.9)	0.074
Liver disease	6 (2.2)	1 (1.6)	1	5 (1.7)	2 (3.6)	0.317	5 (1.9)	2 (2.3)	0.774
Chronic obstructive pulmonary disease (COPD)	33 (12.1)	9 (15.3)	0.519	35 (12)	8 (14.3)	0.639	30 (11.3)	13 (15.1)	0.276
Chronic cognitive deficit	15 (5.5)	8 (13.1)	0.046	22 (7.5)	1 (1.8)	0.146	17 (6.4)	6 (7)	0.802
Regular medication (%)									
ACEi/ARBs	155 (56.8)	32 (52.5)	0.539	168 (57.5)	24 (42.1)	0.032	151 (57.2)	41 (48.2)	0.149
Statins	158 (57.9)	33 (53.2)	0.504	169 (57.7)	29 (50.9)	0.343	152 (56.7)	46 (54.1)	0.6
Immunosuppressive drugs	15 (5.5)	6 (9.7)	0.220	19 (6.5)	2 (3.5)	0.548	15 (5.7)	6 (7.1)	0.642
In-hospital outcomes (%)									
ICU admission	28 (10.1)	27 (42.9)	<0.001				2 (0.7)	56 (65.1)	<0.001
In-hospital death				30 (10.1)	27 (46.6)	<0.001	14 (5.5)	49 (58.3)	<0.001
Hospital LOS, median (IQR)	11 (7–17)	13 (7–22)	0.246	10 (6–15)	23 (15–32)	<0.001	10 (6–15)	17 (9–26)	<0.001
ICU LOS, median (IQR)	14 (12–22)	11 (8–18)	0.031	0	13 (10–20)	1	11	13 (10–21)	0.544
COVID-19 Complications (%)									
Acute respiratory distress syndrome (ARDS)	35 (12.7)	49 (77.8)	<0.001	30 (10.1)	56 (96.6)	<0.001			
Acute kidney injury (AKI)	18 (6.5)	21 (33.3)	<0.001	33 (11.1)	10 (17.2)	0.194	23 (8.6)	20 (23.3)	<0.001
Acute cardiac injury (ACI)	9 (3.3)	22 (34.9)	<0.001	23 (7.8)	8 (13.8)	0.140	11 (4.1)	20 (23.3)	<0.001
Acute liver injury	17 (6.2)	10 (16.1)	0.017	15 (5.1)	13 (22.4)	<0.001	12 (4.5)	16 (18.8)	<0.001
Shock	3 (1.1)	25 (40.3)	<0.001	12 (4.1)	16 (28.1)	<0.001	7 (2.6)	21 (24.7)	<0.001
Bacterial superinfection	47 (17)	36 (57.1)	<0.001	48 (16.3)	36 (62.1)	<0.001	36 (13.5)	48 (55.8)	<0.001
Thrombotic event	17 (6.2)	16 (25.4)	<0.001	22 (7.4)	12 (20.7)	0.002	9 (7.1)	15 (17.4)	0.006
Hemodialysis	2 (0.7)	3 (4.8)	0.047	2 (0.7)	3 (5.2)	0.034	2 (0.8)	3 (3.5)	0.097
Diabetic ketoacidosis (DKA)	3 (1.1)	1 (1.6)	0.564	3 (1)	1 (1.7)	0.514	2 (0.7)	2 (2.3)	0.250
COVID-19 Treatment modalities (%)									
Remdesivir	149 (54.2)	31 (49.2)	0.551	159 (54.1)	35 (60.3)	0.381	146 (54.7)	48 (56.5)	0.773
Corticosteroids	229 (83)	60 (95.2)	0.013	245 (82.8)	58 (100)	<0.001	218 (81.3)	85 (98.8)	<0.001
Antibiotics	231 (84)	63 (100)	<0.001	241 (81.7)	57 (98.3)	0.001	214 (80.1)	84 (97.7)	<0.001
Tocilizumab	45 (16.4)	18 (28.6)	0.023	40 (13.7)	23 (40.4)	<0.001	32 (12)	31 (36.9)	
Anakinra	11 (4)	0	1	11 (3.8)	0	1	11 (4.1)	0	
Oxygen therapy	249 (90.5)	61 (96.8)	0.103	264 (89.5)	57 (98.3)	0.033	236 (88.4)	85 (98.8)	0.003
High flow nasal cannula (HFNC)	14 (5.2)	15 (24.2)	<0.001	15 (5.1)	16 (29.1)	<0.001	8 (3)	23 (27.7)	<0.001
Non-invasive ventilation (NIV)	73 (26.7)	19 (30.2)	0.583	80 (27.2)	13 (22.8)	0.49	66 (24.8)	27 (31.8)	0.206
Type 2 DM chronic treatment regimen (%)									
Antidiabetic tablets	238 (87.5)	51 (82.3)	0.275	250 (85.9)	51 (87.9)	0.683	224 (85.2)	77 (89.5)	0.308
Metformin	123 (45.4)	21 (34.4)	0.119	119 (41)	28 (49.1)	0.259	111 (42.2)	36 (42.9)	0.916
Sulfonylureas	27 (9.9)	7 (11.3)	0.749	29 (9.9)	6 (10.5)	0.891	26 (9.8)	9 (10.6)	0.843
Dipeptidyl peptidase 4 (DPP4) inhibitors	99 (35.9)	32 (50.8)	0.028	105 (35.5)	29 (50)	0.037	91 (34)	43 (50)	0.008
Glucagon-like peptide-1 receptor agonists (GLP1-RA)	34 (12.6)	4 (6.5)	0.171	33 (11.3)	5 (8.9)	0.597	31 (11.8)	7 (8.3)	0.378
Insulin	58 (21.2)	14 (22.2)	0.865	63 (22.2)	8 (13.8)	0.150	59 (22.3)	6 (7.1)	0.235
Insulin plus GLP1-RA	11 (4)	2 (3.2)	1	13 (4.4)	1 (1.8)	0.482	11 (4.2)	3 (3.5)	1
Sodium-glucose cotransporter-2 inhibitors (SGLT2i)	46 (16.7)	6 (9.7)	0.168	45 (15.2)	7 (12.3)	0.569	40 (14.9)	12 (14.1)	0.855

ICU; intensive care unit, ARDS; acute respiratory distress syndrome, T2D; type 2 diabetes, BMI; body mass index, ACEi; angiotensin-converting enzyme inhibitors, ARBs; angiotensin II receptor blockers, IHD; ischemic heart disease, CVA; cerebrovascular accident, CHF; congestive heart failure, ESRD; end-stage renal disease, COPD; chronic obstructive pulmonary disease, LOS; length of stay, AKI; acute kidney injury, ACI; acute cardiac injury, HFNC; high flow nasal canulla, NIV; non-invasive ventilation, DPP4i; dipeptidyl peptidase 4 inhibitors, GLP1-RA; glucagon-like peptide-1 receptor agonists, SGTL2i; sodium-glucose cotransporter-2 inhibitors.The multivariate analysis showed that prior DPP4-inhibitor use is associated with increased odds of in-hospital death (adjusted odds ratio (adj. OR) 2.639, 95% confidence interval (CI) 1.148–6.068, *p* = 0.022). BMI (adj. OR 0.892, 95% CI 0.797–0.999, *p* = 0.048), CVA (adj. OR 5.357, 95% CI 1.308–21.943, *p* = 0.02), and a chronic cognitive deficit (adj. OR 4.163, 95% CI 1.064–16.288, *p* = 0.04) were also associated with in-hospital death. Age and CKD failed to reach statistical significance in the adjusted model (Supplementary Table S1).

## Data Availability

Data available upon request from the corresponding author, and public availability is limited due to privacy and ethical constraints.

## Supplementary Materials

The following supporting information can be downloaded at: https://www.mdpi.com/article/10.3390/microorganisms11061416/s1, Table S1: Univariate and Multivariate Logistic Regression Models of In-Hospital Outcomes; Table S2: Cox proportional regression model for 28-day mortality.
