# Supplementary material for: COVID-19 Outcomes and Diabetes Mellitus: A Comprehensive Multicenter Prospective Cohort Study
Source: Microorganisms. 2023 May 27;11(6):1416. doi: 10.3390/microorganisms11061416 (PMC10300742; doi:10.3390/microorganisms11061416)
Supplement: Supplementary file 1 [file microorganisms-11-01416-s001.zip › microorganisms-2401798-supplementary.pdf]

Supplementary Table S1. Univariate and Multivariate Logistic Regression Models of In-Hospital Outcomes														
	In-hospital death				ARDS				ICU admission					
**Variables**	**OR (95% CI)**	**p value**	**adj. OR (95% CI)**	**p value**	**OR (95% CI)**	**p value**	**adj. OR (95% CI)**	**p value**	**OR (95% CI)**	**p value**	**adj. OR (95% CI) – 1st model**	**p value**	**adj. OR (95% CI) -2nd model**	**p value**
**Male sex**	1.180 (0.68-2.045)	0.556	2.223 (0.887-5.572)	0.088	1.393 (0.852-2.280)	0.187	2.194 (1.031-4.669)	0.041	2.222 (1.218-4.054)	0.009	2.278 (1.022-5.075)	0.044	2.575 (1.163-5.701)	0.020
**Age (years)**	1.069 (1.038-1.100)	<0.001	1.042 (0.998-1.087)	0.064	1.015 (0.995-1.036)	0.143	1.043 (1.006-1.081)	0.021	0.985 (0.963-1.008)	0.193	1.046 (1.005-1.089)	0.026	1.050 (1.008-1.093)	0.018
**BMI (kg/m^2)**	0.892 (0.829-0.96)	0.002	0.892 (0.797-0.999)	0.048	0.983 (0.936-1.032)	0.491	1.007 (0.937-1.081)	0.853	1.021 (0.970-1.075)	0.421	1.038 (0.966-1.116)	0.311	1.048 (0.976-1.125)	0.194
**HbA1c (%)**	1.180 (0.963-1.444)	0.110	1.146 (0.843-1.558)	0.384	1.117 (0.925-1.349)	0.249	1.085 (0.845-1.393)	0.524	1.149 (0.943-1.400)	0.168	1.074 (0.826-1.397)	0.592	1.204 (0.921-1.573)	0.175
**Antidiabetic tablets**	0.662 (0.315-1.394)	0.278			1.490 (0.69-3.216)	0.310			1.195 (0.508-2813)	0.684				
**Years living with Diabetes (years)**	1.02 (0.984-1.057)	0.282			0.975 (0.941-1.010)	0.153	0.941 (0.890-0.995)	0.033	0.943 (0.9-0.987)	0.012	0.918 (0.858-0.982)	0.013	0.935 (0.876-0.998)	0.045
**Metformin**	0.632 (0.354-1.128)	0.120			1.027 (0.625-1.688)	0.916			1.387 (0.785-2.452)	0.260				
**Sulfonylureas**	1.155 (0.478-2.788)	0.749			1.084 (0.487-2.414)	0.843			1.067 (0.421-2.701)	0.891				
**DPP4**	1.846 (1.063-3.205)	0.03	2.639 (1.148-6.068)	0.022	1.945 (1.189-3.183)	0.008	2.507 (1.278-4.916)	0.007	1.819 (1.032-3.207)	0.039	2.524 (1.217-5.232)	0.013		
**GLP1-RA**	0.479 (0.163-1.403)	0.179			0.68 (0.288-1.607)	0.380			0.766 (0.286-2.057)	0.598				
**Insulin**	1.059 (0.547-2.051)	0.865			0.679 (0.357-1.289)	0.237			0.561 (0.253-1.244)	0.155			0.259 (0.074-0.909)	0.035
**Insulin+GLP1RA**	0.794 (0.171-3.676)	0.768			0.845 (0.23-3.102)	0.799			0.385 (0.049-3)	0.362				
**SGLT2i**	0.536 (0.218-1.317)	0.174			0.937 (0.467-1.881)	0.937			0.781 (0.333-1.831)	0.569				
**ACEi/ ARBs**	0.84 (0.481-1.466)	0.539			0.697 (0.427-1.139)	0.150	1.018 (0.411-2.521)	0.970	0.537 (0.302-0.954)	0.034	0.951 (0.350-2.578)	0.921	0.699 (0.259-1.888)	0.480
**Statins**	0.828 (0.476-1.441)	0.505			0.877 (0.537-1.433)	0.600			0.760 (0.430-1.342)	0.344				
**IHD**	2.417 (1.342-4.353)	0.003	1.902 (0.731-4.954)	0.118	1.550 (0.892-2.692)	0.120	1.218 (0.571-2.596)	0.610	1.085 (0.56-2.101)	0.809				
**CVA**	5.058 (2.235-11.443)	<0.001	5.357 (1.308-21.943)	0.02	1.583 (0.687-3.649)	0.281	4.319 (1.124-16.604)	0.033	0.383 (0.088-1.663)	0.200	1.170 (0.191-7.166)	0.865	1.440 (0.245-8.462)	0.687
**Revascularization of any artery**	1.291 (0.498-3.345)	0.599			1.212 (0.516-2.846)	0.659			1.082 (0.395-2.965)	0.879				
**Hypertension**	1.069 (0.57-2.004)	0.835			0.653 (0.386-1.107)	0.114	0.592 (0.226-1.552)	0.286	0.448 (0.249-0.808)	0.008	0.407 (0.147-1.129)	0.063	0.489 (0.179-1.340)	0.164
**CHF**	2.161 (1.094-4.270)	0.027	1.552 (0.485-4.968)	0.459	1.074 (0.542-2.127)	0.838			0.514 (0.195-1.357)	0.179	0.282 (0.072-1.105)	0.069	0.340 (0.087-1.328)	0.121
**Malignancy**	2.780 (1.165-6.631)	0.021	1.093 (0.248-4.822)	0.907	2.103 (0.916-4.831)	0.080	1.379 (0.366-5.195)	0.635	1.241 (0.448-3.439)	0.678				
**ESRD**	3.345 (1.761-6.353)	<0.001	2.226 (0.867-5.718)	0.096	1.483 (0.789-2.789)	0.221	0.925 (0.364-2.350)	0.870	0.837 (0.373-1.879)	0.666				
**Immunosuppresion**	1.843 (0.685-4.959)	0.226			1.261 (0.473-3.359)	0.643			0.522 (0.118-2.308)	0.392				

**Liver disease**	0.733 (0.087-6.205)	0.776			1.274 (0.243-6.692)	0.775			2.111 (0.399-11.163)	0.379				
**COPD**	1.309 (0.59-2.906)	0.508			1.476 (0.73-2.984)	0.279	1.416 (0.571-3.508)	0.453	1.219 (0.533-2.789)	0.639				
**Chronic cognitive deficit**	2.616 (1.056-6.484)	0.038	4.163 (1.064-16.288)	0.040	1.131 (0.431-2.969)	0.802			0.226 (0.03-1.709)	0.150	0.141 (0.014-1.416)	0.096	0.172 (0.018-1.619)	0.124

OR; odds ratio, adj OR; adjusted odds ratio, BMI; Body mass index, DPP4i; Dipeptidyl peptidase 4 inhibitors, GLP1-RA; Glucagon-like peptide-1 receptor agonists, SGTL2i; Sodium-glucose cotransporter-2 inhibitors, ACEi; Angiotensin-converting enzyme inhibitors, ARBs; Angiotensin II receptor blockers, IHD; Ischemic Heart disease, CVA; cerebrovascular accident, CHF; congestive heart failure, ESRD; end stage renal disease, COPD; chronic obstructive pulmonary disease

Supplementary Table S2. Cox proportional regression model for 28-day mortality

	p value	adj. HRs	95,0% CI	
			Lower	Upper
**Male gender**	0,012	2,468	1,222	4,984
**BMI (kg/m²)**	0,250	0,960	0,895	1,029
**Age (years)**	0,003	1,058	1,019	1,099
**Ischemic Heart Disease**	0,070	1,893	0,949	3,774
**Cerebrovascular accident**	0,050	2,379	0,999	5,663
**End-stage kidney disease**	0,160	1,688	0,813	3,507
**DPP4 inhibitors**	0,027	2,014	1,082	3,750
**Congestive Heart Failure**	0,839	0,917	0,397	2,118
**Malignancy**	0,919	1,049	0,419	2,626
**Chronic neurologic deficit**	0,001	4,262	1,759	10,322
**Years since T2D diagnosis**	0,021	0,948	0,905	0,992

Adj. HRs; adjusted hazard ratios, BMI; Body mass index, DPP4i; Dipeptidyl peptidase 4 (DPP4), T2D; Type 2 Diabetes
